# Challenges in nodal peripheral T-cell lymphomas: from biological advances to clinical applicability

**DOI:** 10.3389/fonc.2023.1150715

**Published:** 2023-04-28

**Authors:** Jasmine Zain, Avyakta Kallam

**Affiliations:** Department of Hematology/Hematopoietic Cell Transplantation, City of Hope Medical Center, Duarte, CA, United States

**Keywords:** T cell lymphoma, updated classification, PTCL-NOS, TFH-cell lymphoma, anaplastic large cell lymphoma, novel therapies

## Abstract

T cell lymphomas are a heterogenous group with varying biological and clinical features that tend to have poor outcomes with a few exceptions. They account for 10-15% of all non-Hodgkin lymphomas (NHL), and 20% of aggressive NHL. There has been little change in the overall prognosis of T cell lymphomas over the last 2 decades. Most subtypes carry an inferior prognosis when compared to the B cell lymphomas, with a 5-year OS of 30%. Gene expression profiling and other molecular techniques has enabled a deeper understanding of these differences in the various subtypes as reflected in the latest 5th WHO and ICC classification of T cell lymphomas. It is becoming increasingly clear that therapeutic approaches that target specific cellular pathways are needed to improve the clinical outcomes of T cell lymphomas. This review will focus on nodal T cell lymphomas and describe novel treatments and their applicability to the various subtypes.

## 1 Introduction

A diagnosis of Peripheral T cell lymphomas (PTCL) remains challenging and confusing for physicians as well as patients. This has to do with the rarity of this group of diseases with clinical features that overlap other illnesses and an overall poor prognosis. PTCL are a heterogenous group of lymphomas, arising from the post thymic T lymphocytes. They account for 10-15% of all non-Hodgkin lymphomas (NHL), and 20% of aggressive NHL. The incidence of PTCL in all age groups is 2.1 per 100,000 (1). The recent world health organization (WHO) classification describes over 30 distinct subtypes of T cell lymphomas (2). In 2004, a letter to the editor identified the “large and unexplored problem of T cell lymphomas” (3) and outlined the poor outcome of T cell lymphomas compared to aggressive diffuse B cell lymphomas by publishing a survival curve that showed a 5-year survival of 30% in PTCL vs over 50% for B cell lymphomas with CHOP like therapies only with the exception of anaplastic large cell lymphoma. They pointed out that this was very likely the result of the very aggressive nature of T cell lymphomas and lack of specific therapies for T cell lymphomas. There was no reason to believe that regimens used to treat aggressive B cell lymphomas should work in T cell lymphomas. Since then, there has been a vast increase in our understanding of T cell lymphomas and there have been treatments studied and approved that are specific to T cell lymphomas. These include pralatrexate, romidepsin, belinostat and Brentuximab Vedotin (4–7). Despite this, there has been little change in the overall prognosis of T cell lymphomas over the last 2 decades (8). Most subtypes carry an inferior prognosis when compared to the B cell lymphomas, with a 5-year OS of 30% (9, 10). This is in part due to lack of understanding of subtype specific pathogenesis as well as the challenges of performing prospective clinical trials evaluating therapies in this field of rare diseases.

In recent years, techniques of molecular biology have allowed the exploration of the genomic landscape of various subtypes of PTCL leading to a better understanding of the pathobiology of the diseases, as well as risk stratification and have paved a way for development of targeted molecules. Gene expression profiling (GEP) has been instrumental towards better identifying the subsets of PTCL and identifying prognostic markers. GEP and whole genome sequencing have also recognized distinct epigenetic regulators contributing to the lymphomagenesis (11). This is now leading to development of targeted therapies that are expected to change the treatment paradigms of PTCL. This article with review the current developments in the understanding of nodal PTCL from lymphomagenesis to therapeutic developments.

## 2 Updates in classification of PTCL

The most recent fifth edition of the world health organization (WHO) classification (2) and the international consensus classification of mature lymphoid neoplasms (ICC 2022) (12) now incorporates the recent advances in the understanding of T cell lymphomas. The mature T cell and NK-cell neoplasms are broadly grouped into 9 types, based on the cell of origin, cytomorphology, disease localization and clinical features (2). Broadly classified into nodal T cell lymphomas and extra-nodal leukemic T cell lymphomas, the major subtypes are summarized in **Table 1** alongwith the comparison to the older 2017 classification. A more detailed description of the individual nodal subtypes are described below:

**Table 1:** Outlines the changes made to the nomenclature/diagnostic criteria for nodal PTCL’s.

2022 ICC Classification	2022 WHO classification	2017 updated WHO classification	Comments
Peripheral T-cell lymphoma, not otherwise specified	Peripheral T-cell lymphoma, not otherwise specified	Peripheral T-cell lymphoma, not otherwise specified	Remains a diagnosis of exclusion; Two biological variants - PTCL-TBX21 and PTCL-GATA3 are identified. Cytotoxic EBV-negative cases may represent a distinct subgroup
TFH lymphoma, angioimmunoblastic subtype	Nodal TFH cell lymphoma, angioimmunoblastic-type	Angioimmunoblastic T cell lymphoma	Diagnostic criteria is unchanged. Grouped under nodal T follicular helper cell lymphoma (TFH). New terminology introduced to group lymphomas with similar phenotype and gene expression profiling signatures.
TFH lymphoma, follicular type	Nodal TFH cell lymphoma, follicular-type	Follicular T cell lymphoma	New nomenclature. Diagnostic criteria is unchanged.
TFH lymphoma, NOS	Nodal TFH cell lymphoma, NOS	Nodal peripheral T-cell lymphoma with TFH phenotype	New nomenclature. Diagnostic criteria is unchanged.
Anaplastic large cell lymphoma, ALK positive	ALK positive, Anaplastic large cell lymphoma	Anaplastic large cell lymphoma, ALK-positive	No change in diagnostic criteria
Anaplastic large cell lymphoma, ALK negative	ALK negative, Anaplastic large cell lymphoma	Anaplastic large cell lymphoma, ALK-positive	No change in the diagnostic criteria. Recognized as a heterogenous entity. TP63 rearrangements,
Breast implant associated ALCL	Breast implant associated ALCL	Breast implant associated ALCL	Overexpression of PD-L1 in over half the cases Constitutive JAK-STAT activation by somatic mutations of STAT3, STAT5B, JAK1 and JAK2 and loss-of function mutations of SOCS1 and SOCS3
Primary nodal EBV+ NK/T cell lymphoma	EBV+ve nodal NK and T cell lymphoma	New entity	New distinct entity from PTCL -NOS. Now grouped under EBV+ve positive NK/T cell lymphomas

The table compares the nomenclature differences between the recent classifcations for nodal T cell lymphomas to the 2017 revised WHO classification. 2022 (ICC) International consensus classification of mature lymphoid neoplasms;. 2022 (WHO) World health organization classification of T cell lymphomas. TFH, T follicular helper cell; NOS, not otherwise specified; ALK,anaplastic lymphoma kinase; ALCL, anaplastic large cell lymphoma; EBV, Epstein barr virus.

The various subtypes of peripheral T cells that form the complex immune system leave the thymus as mature T cells after undergoing T cell receptor (TCR) rearrangement, a process that is mediated by a complex array of cytokines and their receptors. The differentiation of these cells is mediated by transcription factors such as FOXP3 for T_regs_, Bcl 6 for T_FH_ Tbet for Th1 and GATA3 for Th2 transcription factors. Each of these cells secrete distinct cytokines, which play a role in the signaling pathways of the immune system and dysregulation of these pathways is thought to play a role in the tumorigenesis. The variability among several types of T cells and their differentiation forms the basis for the heterogeneity noted in PTCL. Gene expression profile (GEP) studies have identified subsets of PTCL’s with molecular signatures similar to the non-neoplastic cells (11, 13–15).

### 2.1 PTCL-Nos

PTCL – NOS is a heterogenous category that does not correspond to any defined subgroup of PTCL and is typically a diagnosis of exclusion. PTCL- NOS is the most common subtype in the Western countries. It affects older patients, with a mean age of diagnosis of 60 years. There is a slight male predilection, with a majority of patients presenting with advanced stage disease. Patients typically present with nodal disease. They may also be extra-nodal involvement, with skin and gastrointestinal tract being the most common extra nodal sites of disease (16). Morphologically, PTCL- NOS demonstrates paracortical infiltrates, with effacement of the normal lymph node architecture. It is associated with expression of CD3, CD4, loss of CD7, CD5. CD30 is expressed in about 32% to 64% of cases with PTCL-NOS (17). Epstein barr virus has been reported to be present in 30% of all PTCL-NOS (16). However, in the recent 5^th^ WHO classification, the EBV+ ve lymphomas are recognized as a distinct entity and are grouped under EBV+ve NK/T cell lymphomas. GEP studies identified two sub-groups within PTCL- NOS (11). One sub-group, designated as PTCL- TBX21 is characterized by expression of TBX21 and target genes such as CXCR3, CCL3 and IL2RB. TBX21 is the master regulator of Th1 cells. PTCL- TBX21 subtype is associated with dysregulated nuclear factor kappa B(NF-kB) pathway (18). The other group, PTCL- GATA3 is characterized by overexpression of GATA3 and its target genes such as CCR4, CXCR7, IL18RA an IK (19). and regulates Th2 differentiation, pointing towards a different cell of origin for these two subtypes. On the other hand, PTCL -GATA3 seen in 33% of the cases and is associated with poor prognosis, irrespective of the TpP53 status. Next generation sequencing shows PTCL- GATA3 to have high genomic complexity, with frequent loss of Tp53, PTEN and CDKNA (18). It is characterized by MYC over-expression and has more cytotoxic features. PTCL- GATA3 subtype is associated with altered PI3 Kinase – mTOR signaling as well (18). Although these are not recognized as distinct subtypes in the WHO classification, they are noted to have different morphological features. PTCL-TBX21 has a more polymorphic background, with reactive inflammatory cells when compared to PTCL -GATA3. PTCL -GATA3 has sheets of medium sized tumor cells with clear cytoplasm and is characterized by a lack of inflammatory background (19).

Epigenetic mutations (described in detail below) are less frequent than in AITL or PTCL with T_FH_ phenotype. TET2 mutations are seen on 38%-49%, DNMT3A (5%-36%) of PTCL-NOS. Mutations in epigenetic regulator genes such as KMT2C, KMT2D, KMT2A could have a possible association with a poor progression free survival and predict responsiveness to HDAC inhibitors, but more studies are warranted. Mutations in the TCT signaling/NF-kB genes have been reported in VAV1, RHOA, CARD11, CD23 and PTPRX (20). In-frame fusion transcripts involving TCR signaling genes have been identified [FYN: TRAF3IP2, KHDRBS1:LCK], which activate signaling pathways downstream of TCR complex and could be a potential target for tyrosine kinase inhibitors. Recurrent mutations in a tumor suppressor gene, FAT1 have been found in 39% of the cases and is associated with a poor prognosis (21).

### 2.2 Nodal T follicular helper cell lymphoma

This new nomenclature was introduced in the 2022 5^th^ WHO classification to group together PTCLS that have a T_FH_ phenotype, overlapping molecular and clinical features. Specific diagnostic criteria have been described that include a primary nodal/systemic process expressing CD4 and have three of the five TFH markers, i.e CD10, BCL6, CD279, CXCL13, and ICOS. The cells also tend to express T cell surface antigens -CD2, CD3 and CD5, which can be detected on flow cytometry or on immunohistochemical staining. The expression of at least two TFH markers (CD10, BCL-6, CD279, CXCL13 and ICOS) are required only for nodal T-follicular helper lymphomas (nTFH), non-otherwise specified (nTFH-NOS); previously designated as Peripheral T-cell lymphoma with follicular helper phenotype (PTCL-TFH). The AITL group and follicular type do not require a threshold cut-off expression of those markers as the morphological architecture is characteristic.

There are three nodal lymphomas which have TFH cell origin that are sub grouped under this category - angioimmunoblastic subtype (previously known as AITL), Nodal TFH cell lymphoma, follicular-type (previously known as follicular T cell lymphoma) and Nodal TFH cell lymphoma, NOS (previously known as Nodal peripheral T cell lymphoma with a T follicular helper phenotype). GEP has been instrumental in differentiating among these subtypes (22).

#### 2.2.1 TFH lymphoma, angioimmunoblastic subtype [TFH lymphoma - AITL subtype]

TFH lymphoma - AITL subtype is commonly seen in older individuals and is characterized by diffuse lymphadenopathy, along with diverse constitutional signs and symptoms. It is often associated with skin rashes, and autoimmune features, including cold agglutinin hemolytic anemia and immune mediated cytopenia’s. Epstein-Barr virus (EBV) positive B immunoblasts are present, but the neoplastic cells do not have EBV (22). AITL exhibits various histological patterns and is often associated with a prominent microenvironment that can obscure the neoplastic cells, atypical B cell proliferations and clonal B cell expansion can be seen. This lymphoma is characterized by effacement of the normal architecture of the lymph node, with diffuse infiltrates of medium sized, atypical lymphocytes with clear cytoplasm Proliferation of arborizing post capillary vessels, is considered one of the hallmarks of this disease. TFH -AITL subtype is most frequently found to have mutations resulting in epigenetic dysregulation and can explain the higher response rates that are seen with epigenetic therapies. TET2 is present in 50-90% of cases, DNMT3A and IDH2 are seen on 20% to 55% of cases (23) An inactivating RHOA mutation can be seen in 70% of TFH lymphoma -AITL subtype and usually co-occurs with TET2 mutations. RHOA_GI7V_ mutated AITL has a higher incidence of B symptoms, splenomegaly, and increased expression of T_FH_ markers. IDH2 _R172_ mutation is associated with a histologically distinct subgroup, with medium to large clear cells and CXCL13 expression (24). Other mutations that are present are CD28, VAV1, PLCG1, STAT3, JAK2 (25).

#### 2.2.2 Nodal TFH cell lymphoma, follicular helper type

FTCL predominantly has a follicular growth pattern and lacks the classical morphologic features of AITL. Morphologically, two distinct growth patterns are recognized: a follicular growth pattern that mimics B-cell follicular lymphoma (FL-like) and a progressive transformation of a germinal centers-like pattern (PTGC-like) In cases with the FL-like pattern, the neoplastic T-cells are arranged in well-defined nodules that lack the morphological features of normal follicles. In PTGC-like cases, the neoplastic T-cells are seen in small aggregates surrounded by small mantle zone B-cells arranged in large, irregular nodules. FTCL shares similar clinical features and mutational profiles with AITL and nodal TFH lymphoma, NOS. phenotype. A t(5;9) (q33;q22) ITK : SYK translocation has been reported in 15% of FTCL, which results in constitutive SYK kinase activation.

#### 2.2.3 Nodal TFH lymphoma, NOS

Nodal TFH NOS phenotype show an infiltrative growth pattern and lack vascular proliferation, without sufficient pathological features to be diagnosed as AITL. Their mutational profile is similar to that of AITL. TET2, DNMT3A, IDH2R172, and RHOAG17V are frequently identified. Mutations in TCR signaling genes, such as PLCG1, PIK3R1, CARD11, CTNNB1, and KRAS have also been reported (23).

### 2.3 Anaplastic large cell lymphoma

The 5^th^ edition of the WHO classification defines ALCL as mature T cell lymphomas characterized by sheets of pleomorphic, large horse-shoe shaped lymphoma cells, with a uniform strong expression of CD30 and often a lack of expression of T lineage markers. There are three distinct subtypes recognized in this classification – ALK negative ALCL, ALK positive ALCL and breast implant associated ALCL (26, 27).

#### 2.3.1 Systemic ALCL

They have a nodal presentation and is classified into ALK+ and ALK-ve ALCL based on the presence of ALK protein detected by IHC (26). The high expression of CD30 has led the path to CD30 directed therapies like Brentuximab Vedotin with high response rates in this subtype (27).

ALK+ve ALCL is seen in younger patients and is associated with good prognosis if the IPI is low at presentation (28, 29). Presence of ALK by IHC is associated with an improved 5-year OS of 79% when compared to 46% for ALK negative patients (29, 30). ALK has several fusion partners, with NPM 1 on chromosome 5 being the most common one (31). ALK gene fusions lead to activation of multiple intracellular transduction pathways, notably the JAK-STAT3, NOTCH and PI3Kinase pathways (32). TP53 mutation is seen in 11% of ALK +ve cases and more frequently seen in younger patients (33). LRP1B gene mutation is reported but does not play a role in the prognosis. NOTCH1 is seen in 20% of patients and has been associated with increased cellular proliferation (34).

ALK negative ALCL nodal lymphomas have all the other morphological and phenotypical features of a CD30 positive cytotoxic ALCL, except ALK expression. They occur in an older age group and in general have a prognosis that is intermediate between ALK+ ALCL and other PTCL histology’s. Emerging molecular data shows substantial heterogeneity, with several subgroups emerging (35). About 70% of Alk-ve ALCLs also express activated STAT3 through tyrosine kinase activity mediated by TYK or ROS1 and mutations of JAK1 and STAT 3 itself. Two recurrent and mutually exclusive rearrangements – DUSP22 and TP63 are seen in ALK-ve ALCL (18). DUSP22-R can be seen in 30% of the cases, is thought to have tumor suppressor function, has several distinct clinicopathological and molecular features (36). Morphologically, ALK negative DUSP22- R is characterized by monomorphic medium sized cell population with increased doughnut shaped cells, lack of JAK-STAT3 pathway activation, absence of PD-L1 expression, characteristic GEP signature (37). Although in the 2022 ICC classification, ALK-negative ALCL with DUSP22-R is considered as a subtype of ALK-negative ALCL, the WHO classification does not recognize DUSP22 -R ALK negative ALCL. (12) DUSP-22 R in general is considered to have favorable prognosis, with outcomes comparable to ALK+ve ALCL, however recent studies have also reported poor outcomes in patients with DUSP22-R (38). Fluorescence *in situ* hybridization (FISH) for DUSP22-R is recommend in ALK negative ALCL to identify the subtype for prognostic purposes. Testing for JAK2- rearrangements may help not only in identification but may also have therapeutic implications (39). However, further studies are needed before FISH can be used in routine clinical practice.

TP63 mutations are associated with a poor prognosis, with a 5 year survival of 17%. GEP studies have shown enrichment of IRF4 and MYC signature as well as proliferation of mTOR gene signatures in ALK-ve ALCL (35, 35). There is low expression of genes involved in TCR signaling and a high expression of *CD30* and its associated genes (*TNFRSF8, BATF3*, and *TMOD1*). There are altered responses to proapoptotic signals as well as Treg and TAM mediated immune dysregulation. PD-1 overexpression is also present in some DUSP22-R negative ALCL. This may provide further therapeutic opportunities.

#### 2.3.2 Breast implant associated ALCL

Breast implant associated ALCL is typically a non-invasive neoplasm occurring in association with textured surface breast implants (40). It is an entity considered distinct from ALK-ve ALCL and is associated with an excellent prognosis. GEP show consistent absence of ALK, DUSP22 and TP63 rearrangements (41). Invasion of adjuvant structures is less common and associated with a poor prognosis. Studies have shown the pathogenesis to be mediated by the TH2 inflammatory cells, subsequent immune evasion. Certain bacterial pathogens such as *Ralstonia* have also been implicated (42). PD-L 1 overexpression is seen in over 50% of the cases. Somatic mutations of STAT3, STAT5B, JAK1 and JAK 2 resulting in constitutive activation of the JAK-STAT pathways, loss of function mutations of SOCS1 and SOCS have also been reported (43). Treatment consists of removal of the implant. Systemic therapy is reserved only for extracapsular spread (40).

### 2.4 EBV positive T and NK cell lymphoma

EBV positive T and NK cell lymphoma was previously classified under the PTCL-NOS. It is now recognized as a distinct entity in both the WHO classification and the 2022 ICC classification (12). It is seen more commonly among East Asians, with patients presenting with advanced lymphadenopathy with or without extra nodal involvement, B symptoms. EBV positive T/NK cell lymphoma is an aggressive disease, with a median survival of 1.5 months (44). Morphologically, diffuse infiltrate of uniform appearing medium to large, EBV+ve cells are noted. Absence of angioinvasion and necrosis are the characteristic features and are used for identification of this lymphoma. The lymphoma shows increased expression of PD-L1, demonstrates frequent mutations of TET2 and PI3 Kinase (45).

## 3 Therapeutic advances

Improved understanding of the tumor biology has not only led to more accurate diagnosis, risk stratification and prognosis, but has also identified critical pathways, which can potentially be altered to improve treatment outcomes. Novel agents targeting epigenomic mutations, cell cycle signaling pathways, tumor microenvironment and cell surface receptors are being used with promising results. **Table 2** summarizes some of the potential novel agents and their targets that are being used and are under investigation. These will be discussed in detail below

**Table 2:** Novel agents for treatment of PTCL and their targeted pathways.

Target	Novel agents
Epigenomic mutations	HDAC inhibitors Hypomethylating agents EZH2 inhibitors
Signaling pathways/Kinase directed therapies	PI3Kinase inhibitors Janus kinase pathway inhibitors SYK pathway inhibitors Aurora kinase inhibitors ALK inhibitors
Cell surface receptors	CD 30 inhibitors CD 25 directed therapies
Tumor microenvironment	Immunomodulatory agents Check point inhibitors Anti-CD 47 agents
Non cell signaling pathways inhibition	Antiapoptotic therapies Farnesyl transferase inhibitors

### 3.1 Epigenetic mutations and targeted therapies

Changes in epigenetic signaling and DNA methylation have been described in T cell lymphoma (46). Epigenetic modifiers such as TET2, DNMT1, DNMT3A, 3B and TDH2 are implicated in T cell lymphoma pathogenesis. TET2 mutations result in cytosine demethylation and repress gene transcription (47). IDH2 is a specific isoform of the krebs cycle isocitrate dehydrogenase and the mutated enzyme catalyzes the conversion of alpha ketoglutarate (TET2 co-factor) to 2- hydroxyglutarate. Although IDH2 is not an epigenetic modifier, its alterations affect epigenetic modifications downstream on the TET2 pathway. TET2 mutations are loss of function mutations and have been implicated in the pathogenesis of AITL and T_FH_ derived PTCL (70-80% of cases), ALK- negative ALCL (33% of cases). DNTM3A mutations are seen in about 11-33% of patients with PTCL, the mutations frequently co-exist with TET2 mutations, resulting in suppression of transcription (47). These mutations are more commonly seen in AITL. Both TET2 and DNMT3A mutations occur during early hematopoietic stem cell maturation. IDH2 mutations are more commonly seen in AITL and their occurrence correlates with T_FH_ GEP signatures (24). EZH2 (enhancer of zeste homolog 2) has found to be mutated and overexpressed in several subtypes of PTCL (48). In ATLL, NF-kB plays a critical role in expression of EZH2.

Mutations in EZH2 result in transcriptional silencing, and inhibition of EZH2 in PTCL results in cell growth arrest *via* gene upregulation (48). KMT2D (mixed lineage leukemia 2) encodes histone methyltransferase, with gene mutations seen in 25% of patients with AITL and 36% in PTCL- NOS. Valemetostat is a dual inhibitor of EZH2 and EZH1 and is thought to increase the gene expression of pro-apoptotic and tumor suppressor genes by altering histone methylation. In a phase I study, 45 patients with rel/ref PTCL were treated with single agent valemetostat, with an ORR of 48%. The side effects observed were mainly thrombocytopenia (59%) and dysgeusia (51%) (49). A phase II study evaluating the efficacy of valemetostat in rel/ref PTCL is ongoing, with early results suggesting it to be an effective agent in rel/ref PTCL (50).

Histone deacetylate inhibitors (HDACI) such as romidepsin and belinostat are approved for relapsed PTCL (51–53). Given that the epigenomic dysfunction is seen to a greater extent in AITL and PTCL – FH subtype, the HDACS seem to have a higher impact in these subtypes. The ORR of romidepsin in AITL is 30% when compared to an ORR of 25% across all subtypes of PTCL. Studies have shown a response rate of 45% in AITL (52). Hypomethylating agents such as 5- azacytidine and decitabine, inhibit DNMT and are being evaluated in AITL (54). A study by Delarue et al. evaluated the efficacy of 5-azacitidine in 19 patients with relapsed/refractory PTCL. Twelve patients had AITL (54). The ORR was 75% in patients with AITL when compared to 15% on other subtypes. It is to be noted that responding patients had a TET2 mutation. Real world data showed an ORR of 40% with 5-azacitdine among patients with heavily pretreated rel/ref AITL (55).

A study evaluating romidepsin and 5 azacytidine in 25 patients with relapsed/refractory PTCL had response rates of 61% and a CR of 48% (56). A retrospective study evaluating real-world data of 26 patients reported an ORR 73% and a CR 53%. Most common mutations found were TET2, RHOA, IDH2 and DNMT3A (57). A phase II study treated patients with 5-azacitidine and CHOP as first line therapy for PTCL (58). This study prioritized patients with TFH subtype of PTCL and enrolled 17 patients with TFH nodal lymphoma, 3 patients with PTCL-NOS and 1 patient with ATLL. Upon completion of treatment, the CR was 76.5% for all patients and 86.7% for patients with TFH nodal lymphoma. The 1-year PFS was also superior at 61% for the TFH lymphoma subtype when compared to 57% for all patients. NGS showed that TET2 mutations were associated with a significantly higher CR rate, favorable PFS and OS (58). The final efficacy data from this study is pending, but the preliminary data appears promising for consideration of such biomarker guided therapies in the front-line setting.

Patients with T_FH_ subtype had response rates of 80%, median progression free survival of 20.6 months. The responders also had a higher average number of DNMT mutations. IDH 2 mutations with substitution at R172 noted in TFH -AITL subtype could be a potential target. A phase Enasidenib, a novel IDH 2 inhibitor is being evaluated in patients with IDH-2 mutated TFH- AITL (NCT02273739) (59).TET- selective small molecular inhibitors (TETi 76) (60), IDH2 specific inhibitors for R172 codon noted in TFH- AITL are also being explored (61).

### 3.2 Signaling pathways

Dysregulated signaling pathways are being evaluated to find novel therapeutic targets. RHOA gene belongs to the Rho family of small GTPases, a group of Ras- like proteins involved in intracellular signaling (62). Gain of function mutations of the RHOA gene drive the T_FH_ differentiation, NFAT signaling activation, augment the PI3 kinase- AKT- mTOR signaling pathway, resulting in increased cell proliferation and transformation. Gain of function mutations are seen in up to 70% of AITL, 15% ATLL and are associated with cell proliferation. Agents targeting the kinase pathways could potentially be more efficacious in patients who have the RHOAG12V mutation. PI3K inhibitors such as duvelisib, multi-kinase inhibitors such as dasatinib have been proposed as therapeutic options (63). Current studies targeting these pathways are described below.

### 3.3 Kinase targeted therapies

#### 3.3.1 PI3kinase inhibitors

PI3kinases are intracellular signaling molecules and are critical for growth and differentiation of lymphocytes. They occur in 4 isoforms – alpha, beta, gamma, delta (64). The gamma and delta subtypes are involved in adaptive immune response and are preferentially expressed in leucocytes. Targeting PI3K – gamma delta pathway has emerged as a promising treatment option for PTCL. Tenalisib and Duvelisib have been evaluated in patients with relapsed/refractory PTCL. A phase I study evaluated duvelisib (oral gamma delta specific PI3Kinase inhibitor) in heavily pretreated 16 patients with relapsed/refractory PTCL and showed an ORR of 50%, with an OS of 8.4 months (65). Grade 2 or higher toxicities included elevated liver function tests (35%), pyrexia (37%), cough (34%), cytopenia’s (24%). This prompted a larger phase II dose finding/expansion study [PRIMO NCT03372057] (66). The preliminary data was consistent with the phase I study, with an ORR of 49%, CR 34% and a median duration of response of 7.7 months (67). Tenalisib, an oral dual PI3Kinase gamma/delta inhibitor was evaluated in 28 patients with heavily treated relapsed/refractory PTCL. Preliminary data shows a response rate of 45%, with a median duration of 4.1 months (68). The toxicity profile is similar to duvelisib. Several combinations with PI3Kinase inhibitors are also being evaluated. Duvelisib and romidepsin have been studied in combination with an ORR of 50%. This combination had lower rates of grade 3/4 transaminitis (8%) when compared to single agent duvelisib (40%), suggesting a potential immunomodulatory effect of romidepsin on PI3Kinase associated transaminitis (69).

Based on the preliminary data and the relatively safe toxicity profile, PI3 Kinase inhibitors are being studied in a combination with immune checkpoint blockade (copanlisib plus pembrolizumab, NCT02535247), with chemotherapy (copanlisib + gemcitabine, NCT03052933. Upfront studies, combining chemotherapy with PI3K inhibitors are ongoing (A051902).

a) Janus Kinase Pathway

The Janus kinase family proteins (JAK1, JAK2, JAK3, TYK2) play a role in mediating immune responses. Downstream to the JAK proteins are STAT family of transcription factors, which regulate the effects of JAK. Activating mutations and gene fusion resulting in activated JAK-STAT pathway signaling have been reported in several subtypes of PTCL. A phase II study of ruxolitinib in 53 patients with relapsed/refractory PTCL reported an ORR of 29% among patients with activating JAK/STAT mutations (70). Response rates were higher in patients with PTCL-T _FH_ subtype and in AITL – 44%, large granular leukemia 75%. The commonly reported grade 3 or higher side effects were cytopenia’s. Golidoctinib, is an oral JAK1 specific inhibitor, is being evaluated in patients with rel/ref PTCL. A phase I/II study (NCT04105010), study enrolled 51 patients with rel/ref PTCL and reports a response rate of 43%. The drug was well tolerated, with grade 3 or higher neutropenia was seen in 29% of the patients, thrombocytopenia seen in 10% (Kim, Yoon et al. 2022).

b) SYK pathway

*SYK* encodes a tyrosine kinase protein that regulates PI3K downstream signaling pathway (71). It is normally expressed on the B cells, but not on the T cells. Aberrant expression of SYK is noted in about 90% of the T cells. Cedulatinib, a JAK/SYK inhibitor was evaluated in 60 patients with PTCL, with an ORR of 35% (72). The response rates in patients with PTCL-T_FH_ type and AITL was 55%. Most common adverse events included elevated lipase, diarrhea, cytopenia’s. TAK-659, a novel SKY inhibitor is currently in development, with preclinical studies showing promising results in non-Hodgkin lymphomas. [NCT-3357626, NCT02000934, NCT03742258]

c) Aurora A kinase pathway

Aurora Kinase is a protein kinase that mediates spindle formation during mitosis and is overexpressed in PTCL. Alisertib is an oral aurora kinase A inhibitor, which showed preclinical activity in PTCL (73). A phase III randomized study of alisertib in rel/ref PTCL showed an ORR 33% (74). However, there was lack of benefit in progression free survival when compared to the control arm and this trial was terminated early.

d) Anaplastic Lymphoma Kinase

As ALCL is divided based on the presence of translocation that fuses the anaplastic lymphoma kinase (ALK) gene to NPM, ALK inhibitors have been evaluated in patients with ALK + ALCL. Crizotinib, an ALK inhibitor is FDA approved for treatment of pediatric patients with rel/ref ALK+ALCL (75). The approval was based in a single arm trial of crizotinib in 26 patients, aged <20 years with relapsed/refractory ALCL. The ORR was 88%. A phase I study evaluated 9 adult patients with heavily pretreated rel/ref ALK+ ALCL (76). All patients experienced complete responses to therapy. There is an ongoing study evaluating ceritinib, a second generation ALK inhibitor, with promising preliminary data (77). Alectinib, a second generation ALK inhibitor has also been evaluated, with promising results. Crizotinib is being evaluated in combination with cytotoxic chemotherapy in patients as a part of front-line therapy in ALK+ALCL [NCT01979536]. *In vitro* studies have shown synergistic activity with crizotinib and brentuximab vedotin (78) and clinical studies are being designed evaluating this combination.

## 4 Specific targeted therapies

### 4.1 CD30 directed therapy

The tumor necrosis family receptor CD30 is universally expressed in ALCL irrespective of the ALK status. It is variably expressed in other subtypes and can be present in 58-64% of PTCL, 43-63% AITL, 55% ATLL. Brentuximab vedotin (BV) is an antibody drug conjugate, composed of an antiCD30 monoclonal antibody linked to monomethyl auristatin E, a microtubule inhibitor (79). BV was first evaluated in patients with rel/ref ALCL, with an ORR of 86% (27). BV also evaluated in patients CD30+ PTCL, with an ORR of 41% and a median PFS of 6.7 months (80). Based on the encouraging results from these studies, ECHELON -2, a randomized double blinded study randomized 226 patients with newly diagnosed PTLD, with at least 10% CD30 expression to receive either BV in combination with cyclophosphamide, vincristine and prednisone (A+CHP) or cyclophosphamide, vincristine, prednisone, doxorubicin (CHOP) (5). Combining BV to chemotherapy proved superior to chemotherapy alone, with a median PFS of 48 months v.s 21 months of CHOP. The hazard ratio was 0.71 indicating a 29% reduction in the risk of relapse. Five-year follow up shows the durability of the responses, with a PFS of 51% with the A+ CHP arm when compared to 43% with the CHOP arm (81). The OS was 70% with the A+CHP arm and 61% with the CHOP arm. The benefit in PFS and OS was seen across all subtypes of PTCL. This is a pivotal study that led to the approval of A+ CHP as front-line therapy for CD30+ PTCL. Other combinations are being evaluated including the combination of BV with CHEP s upfront therapy and other agents like gemcitabine (ASH abstract).

### 4.2 CCR4

CC chemokine receptor 4 (CCR4) promotes memory T cell homing to peripheral tissues and is preferentially expressed by Th2 and regulatory T cell subsets. CCR4 is predominantly expressed in ATLL. CCR4 is also expressed in about 30–65% of other PTCL cells, including a high expression (65%) in ALK -ve ALCL, variable expression (40%) in PTCL- NOS, AITL and transformed MF (82). Mogamulizumab is a humanized, IgG1 monoclonal anti CCR4 antibody. It has a direct cytotoxic effect on CCR-4 positive lymphoma cells *via* ADCC and depletes the regulatory T cell, resulting in an antitumor activity. It is approved for CCR4+ rel/ref ATLL in Japan based on a phase 2 study that reported a response rate of 50%, with median PFS and OS of 5.2 and 13.7 months (83). In the US, it is approved for patients with cutaneous T cell lymphoma. The main side effects are skin reactions seen in up to 63% of patients, infusion reactions and hematologic toxicity.

#### 4.3 CD25 directed therapies

T-cell activation (CD 25) is the alpha subunit of the heterotrimeric IL-2 receptor. CD25 is seen in upto 40-50% of all PTCL’s (84). As it is differentially expressed on malignant T cells, this has been evaluated for a potential target. Antibody drug conjugates towards CD25 have shown promising results. Camidanlumab tesitine, a CD 25 antibody conjugated to cytotoxic pyrrolobenzodiazepine dimer has shown promising results in relapsed PTCL. Responses correlate with CD25 expression (85). Camidanlumab tesitine is currently accruing patients in phase II study, with promising responses seen in patients with hodgkin lymphoma. Radiolabeled antibodies such as Y^90^ labeled basiliximab are being evaluated in combination with cytotoxic chemotherapy in PTCL directed transplant regimens (86).

## 5 Altering the tumor microenvironment

A three-signal pathway is thought to contribute to T cell lymphomagenesis – (i) antigenic stimulation through TCR engagement – signal 1 (ii) co-stimulatory signals from non- MHC surface receptors -signal 2, (iii) cytokine signals through paracrine stimulation – signal 3 (14). Both type 2 and type 3 signals are mediated by non-neoplastic T cells, highlighting the role the tumor microenvironment plays in this disease (14). The tumor cells cause modulation of the microenvironment and decrease the tumor immunogenicity and in doing this, promote their survival (14). The neoplastic T cells produce several soluble cytokines such as IL-5, IL-13 and VEGF and other immune regulatory molecules. The IL-13 recruit eosinophilic granulocytes from the blood stream. These eosinophils secrete high amounts of IL-10, IL-4, which sustain macrophage differentiation into a tumor favoring subtype. These differentiated macrophages express PD-L1 and produce several angiogenetic and immune-modulatory soluble mediators which facilitate tumor immune escape from antitumor cytotoxic T cells. PTCL also recruits non-neoplastic Tregs lymphocytes which dampen the antitumor immune responses (87).

Therefore, targeting the tumor microenvironment may provide a therapeutic opportunity.

### 5.1 Immunomodulatory agents

Lenalidomide acts by modulation of substrate specificity of CRL4^CRBN^ ubiquitin ligase complex and in doing so, induces degradation of protein targets that mediate immune responses such as interleukin production and NK/T cell proliferation (88). By doing so, lenalidomide decreases cell proliferation and enhances NK cell cytotoxicity. Lenalidomide has being investigated in PTCL, both as a single agent and in combination therapy. A phase 1/2 study reported an ORR of 22% in 54 patients with Rel/Ref PTCL (89). The median PFS was 2.5 months. Response rates were comparable across all subgroups. A multicenter trial (EXPECT) reported a similar response rate of 22% with 11% CR, mostly in patients with AITL (90). While single agent activity is low, lenalidomide is being studied in combination regimens in T–cell Lymphomas. The combination of CHOEP and lenalidomide as an upfront treatment resulted in an ORR of 48% in a phase 2 study in 40 patients; however, there was significant toxicity (91). Romidepsin and lenalidomide has been combined safely; the phase 1/2 study of 21 patients produced a response rate of 53% with acceptable hematologic toxicity (92). The combination is now being evaluated in an upfront study of elderly patients with PTCL. The combination of Romidepsin, lenalidomide and carfilzomib resulted in an ORR of 50% in a phase 1/2 trial of 20 patients (93). Lenalidomide is being studied in combination with BV and with immune check point inhibitors such as anti-PD1 antibody durvalumab (NCT03011814) in cutaneous T cell lymphomas.

### 5.2 Checkpoint inhibitors

Use of checkpoint inhibitors in T cell malignancies poses concerns as although there is an anti-tumor effect on the cytotoxic T cells, there could also be an unintended blocking of PD-1 driven tumor suppression maintained by the PD-L1 expressed on the antigen presenting cells. There have been reports of rapid progression in patients with ATLL when treated with nivolumab (94). PD-1 inhibitors have been used in ENKL, with response rates of 100% with pembrolizumab. The EBV viral proteins upregulate the PD-1 receptor levels, which is thought to be the reason for superior response rates with PD-1 inhibitors when compared to.

Pembrolizumab has been used in patients with relapsed/refractory cutaneous lymphomas, with response rates of 38% (95). However, these responses have been short-lived. The use of checkpoint inhibitors in PTCL is currently limited to clinical trials.

### 5.3 Anti CD47 antibodies

CD47 is a cell surface regulator of phagocytosis and is mediated by macrophages and dendritic cells. Increased expression of CD47 results in an inhibitory signal for phagocytosis. Inhibition of CD47 signaling can promote macrophage phagocytosis of tumor cells (96). Anti CD47 directed antibodies are being used in PTCL, with promising results.

## 6 Cellular therapy

Chimeric antigen receptor T cells, targeting CD19 have significantly improved outcomes in B cell lymphomas. CAR-T therapy has since been expanded into treatment of other hematological malignancies. Studies have been ongoing to determine if CAR-T cells could be used to treat T cell lymphomas.

Designing CAR-T cells to target T cell neoplasms has been challenging due to the following complications. CAR-T fratricide, due to endogenous expression of T cell antigens that CAR-T cells are designed against, limits T cell expansion (97). Preclinical studies are ongoing, investigating manipulating the CAR antigen target to bypass the fratricide. T cell aplasia is a potential complication. Unlike B cell aplasia that is encountered with CD 19 CAR’s, T cell aplasia has a risk of permanent immunosuppression, resulting in life threatening infections. CAR-T cells targeting CD 30 are being developed, with pre-clinical studies showing good cell expansion and cytotoxic activity against PTCL xenograft tumors (98). CAR T cells targeting CD4, CD5, CD7 are also being developed. Allogenic T cells and use of alternate cell sources such as CAR-macrophages are also in early phase clinical trials.

## 7 Non- cell signaling Kinase/non epigenetic therapies

### 7.1 Anti-apoptotic therapies

Apoptosis is regulated by BCL2 family of proteins, which function by counter balancing pro and antiapoptotic members, MCL1 and BCL2. High levels of MCL 1 expression have been described in PTCL and inhibition of this pathway is associated with reduced cell survival (99). Koch et al. described MCL1 dependent PTCL xenograft model where MCL 1 inhibition was synergistic with multiagent chemotherapy and improved survival (100). Based on this data, several studies, with agents targeting MCL1 and BCL2 in patients with rel/ref PTCL are ongoing (PRT1419 -NCT04543305; AMG 397 – NCT03465540, venetoclax – NCT03534180).

### 7.2 Farnesyltransferase inhibitors

CXCL12 expression has shown to have a prognostic significance in patients with PTCL. Tipifarnib, a farnesyltransferase/CXCR4 inhibitor has shown to downregulate CXCL12 secretion in stromal cultures (101). CXCL12 can be expressed in up to 50% of AITL. This drug is being selectively investigated in patients with CXCL 12+ PTCL- NOS or AITL. A phase 2 study evaluated the role of tipifanib heavily pretreated patients with AITL or PTCL- NOS (102). In the AITL cohort, there were 11 evaluable patients with an OR of 45%. Among the 3 evaluable CXCL12+ PTCL patients, one patient had a PR and 2 patients had SD to therapy. CXCL 12 expression was found to correlate with favorable outcome to treatment (102). Pre-treatment high CXCL12 expression had 90% sensitivity and 93% specificity to accurately predict a treatment response. The primary toxicities were thrombocytopenia and neutropenia.

## 8 Conclusions

How best to incorporate these novel agents into the existing treatment regimens is both exciting and challenging. Given the relatively low and short-lived response rates with single agent therapy, combination therapies are being explored. For frontline therapy, combination chemotherapy with addition of BV in CD30+ patients have become the current standard of care. Consolidation autologous stem cell transplant is recommended for eligible patients with PTCL, other than ALK+ve ALCL. Treatments combining novel agents such as romidepsin/azacytidine with cytotoxic chemotherapy are being explored. In the rel/ref setting, trials combining novel agents such as copanlisib+ romidepsin, durvalumab+ lenalidomide are being evaluated.

Clinical trials incorporating GEP to tailor treatments are warranted. An umbrella study in Japan is evaluating the efficacy and safety of targeted therapies guided by molecular subtypes in patients with rel/ref PTCL [NCT05559008]. This study is incorporating next generation sequencing to identify the molecular subtypes and interventions are being modified accordingly. The results of these study could potentially be practice changing. More such studies incorporating the molecular advances are warranted. The future for PTCL is promising. Recent advances in molecular and genomic profiling of PTCL has provided a unique insight into the molecular pathways and in the development of new therapeutic agents. Further studies are needed to tailor the treatments based on genomic markers in both front line and relapsed settings. Development of markers to define response to these agents and to predict patterns of treatment failure will help advance the field immensely.

## Author contributions

JZ contributed to manuscript preparation, editing, submission. AK contributed to manuscript preparation. All authors contributed to the article and approved the submitted version.

## References

[1] Swerdlow SH. Who classification of tumours of haematopoietic and lymphoid tissues. Int Agency For Res On Cancer (2017).

[2] Alaggio R Amador C Anagnostopoulos I Attygalle AD Araujo IBDO Berti E. The 5th edition of the world health organization classification of hematolymphoid tumours: Lymphoid neoplasms. Leukemia (2022) 36(7):1720–48. doi: 10.1038/s41375-022-01620-2 PMC9214472 35732829

[3] Armitage J Vose J Weisenburger D. Towards understanding the peripheral T-cell lymphomas. Ann Of Oncol (2004) 15(10):1447–9. doi: 10.1093/annonc/mdh409 15367401

[4] O'connor OA Pro B Pinter-Brown L Bartlett N Popplewell L Coiffier B. Pralatrexate in patients with relapsed or refractory peripheral T-cell lymphoma: Results from the pivotal propel study. J Of Clin Oncol Off J Of Am Soc Of Clin Oncol (2011) 29(9):1182–9. doi: 10.1200/JCO.2010.29.9024 PMC3083873 21245435

[5] Horwitz S O'connor OA Pro B Illidge T Fanale M Advani R. Brentuximab vedotin with chemotherapy for Cd30-positive peripheral T-cell lymphoma (Echelon-2): A global, double-blind, randomised, phase 3 trial. Lancet (2019) 393(10168):229–40. doi: 10.1016/S0140-6736(18)32984-2 PMC6436818 30522922

[6] Piekarz RL Frye R Prince HM Kirschbaum MH Zain J Allen SL. Phase 2 trial of romidepsin in patients with peripheral T-cell lymphoma. Blood (2011) 117(22):5827–34. doi: 10.1182/blood-2010-10-312603 PMC3112033 21355097

[7] Foss F Advani R Duvic M Hymes KB Intragumtornchai T Lekhakula A. A phase ii trial of belinostat (Pxd 101) in patients with relapsed or refractory peripheral or cutaneous T-cell lymphoma. Br J Of Haematology (2015) 168(6):811–9. doi: 10.1111/bjh.13222 25404094

[8] Chihara D Fanale MA Miranda RN Noorani M Westin JR Nastoupil LJ. Survival outcome of patients with relapsed/refractory peripheral T-cell lymphoma-not otherwise specified and angioimmunoblastic T-cell lymphoma. Br J Haematol (2017) 176:750–8. doi: 10.1111/bjh.14477 PMC5836501 27983760

[9] Vose J. International T-cell lymphoma project. international peripheral T-cell and natural Killer/T-cell lymphoma study: Patholegy findings and clinical outcomes. J Clin Oncck (2008) 26:4124–30. doi: 10.1200/JCO.2008.16.4558 18626005

[10] Wang SS Vose JM. Epidemiology and prognosis of T-cell lymphoma. T-Cell Lymphomas. Springer (2013), 25–39. doi: 10.1007/978-1-62703-170-7_2

[11] Iqbal J Liu Z Deffenbacher K Chan WC. Gene expression profiling in lymphoma diagnosis and management. Best Pract Res Clin Haematology (2009) 22(2):191–210. doi: 10.1016/j.beha.2009.05.001 19698928

[12] Feldman AL Laurent C Narbaitz M Nakamura S Chan WC De Leval L. Classification and diagnostic evaluation of nodal T-and nk-cell lymphomas. Virchows Archiv (2022) 482(1):265–79. doi: 10.1007/s00428-022-03412-6 36210383

[13] Wilcox RA. A three-signal model of T-cell lymphoma pathogenesis. Am J Of Hematol (2016) 91(1):113–22. doi: 10.1002/ajh.24203 PMC4715594 26408334

[14] Pizzi M Margolskee E Inghirami G. Pathogenesis of peripheral T cell lymphoma. Annu Rev Of Pathology: Mech Of Dis (2018) 13:293–320. doi: 10.1146/annurev-pathol-020117-043821 29414251

[15] Fiore D Cappelli LV Broccoli A Zinzani PL Chan WC Inghirami G. Peripheral T cell lymphomas: From the bench to the clinic. Nat Rev Cancer (2020) 20(6):323–42. doi: 10.1038/s41568-020-0247-0 32249838

[16] Broccoli A Zinzani PL. Peripheral T-cell lymphoma, not otherwise specified. Blood J Of Am Soc Of Hematol (2017) 129(9):1103–12. doi: 10.1182/blood-2016-08-692566 28115372

[17] Karube K Kakimoto Y Tonozuka YO Ohshima K. The expression of Cd30 and its clinic-pathologic significance in peripheral T cell lymphoma. Expert Rev Of Hematol (2021) 14(8):777–87. doi: 10.1080/17474086.2021.1955344 34263699

[18] Heavican TB Bouska A Yu J Lone W Amador C Gong Q. Genetic drivers of oncogenic pathways in molecular subgroups of peripheral T-cell lymphoma. Blood J Of Am Soc Of Hematol (2019) 133(15):1664–76. doi: 10.1182/blood-2018-09-872549 PMC6460420 30782609

[19] Amador C Greiner TC Heavican TB Smith LM Galvis KT Lone W. Reproducing the molecular subclassification of peripheral T-cell lymphoma–nos by immunohistochemistry. Blood (2019) 134(24):2159–70. doi: 10.1182/blood.2019000779 PMC6908831 31562134

[20] Piccaluga PP Fuligni F De Leo A Bertuzzi C Rossi M Bacci F. Molecular profiling improves classification and prognostication of nodal peripheral T-cell lymphomas: Results of a phase iii diagnostic accuracy study. J Of Clin Oncol Off J Of Am Soc Of Clin Oncol (2013) 31(24):3019–25. doi: 10.1200/JCO.2012.42.5611 23857971

[21] Laginestra MA Cascione L Motta G Fuligni F Agostinelli C Rossi M. Whole exome sequencing reveals mutations in Fat1 tumor suppressor gene clinically impacting on peripheral T-cell lymphoma not otherwise specified. Modern Pathol (2020) 33(2):179–87. doi: 10.1038/s41379-019-0279-8 PMC6994417 31028364

[22] Dobay MP Lemonnier F Missiaglia E Bastard C Vallois D Jais J. Integrative clinicopathological and molecular analyses of angioimmunoblastic T-cell lymphoma and other nodal lymphomas of follicular helper T-cell origin. Haematologica (2017) 102(4):E148–51. doi: 10.3324/haematol.2016.158428 PMC5395128 28082343

[23] Lemonnier F Couronné L Parrens M Jaïs J Travert M Lamant L. Recurrent Tet2 mutations in peripheral T-cell lymphomas correlate with tfh-like features and adverse clinical parameters. Blood J Of Am Soc Of Hematol (2012) 120(7):1466–9. doi: 10.1182/blood-2012-02-408542 22760778

[24] Wang C Mckeithan TW Gong Q Zhang W Bouska A Rosenwald A. Idh2 R172 mutations define a unique subgroup of patients with angioimmunoblastic T-cell lymphoma. Blood J Of Am Soc Of Hematol (2015) 126(15):1741–52. doi: 10.1182/blood-2015-05-644591 PMC4600014 26268241

[25] Willemsen M Hamid A M. W B. And Zur Hausen A. Mutational heterogeneity of angioimmunoblastic T-cell lymphoma indicates distinct lymphomagenic pathways. Blood Cancer J (2018) 8(1):1–4. doi: 10.1038/s41408-017-0047-2 29339730 PMC5802554

[26] Hafeez S Cushman-Vokoun AM. Molecular advances in nodal peripheral T-cell lymphoma. Adv In Mol Pathol (2022) 5(1):51–8. doi: 10.1016/j.yamp.2022.05.002

[27] Pro B Advani R Brice P Bartlett NL Rosenblatt JD Illidge T. Brentuximab vedotin (Sgn-35) in patients with relapsed or refractory systemic anaplastic Large-cell lymphoma: Results of a phase ii study. J Of Clin Oncol Off J Of Am Soc Of Clin Oncol (2012) 30(18):2190–6. doi: 10.1200/JCO.2011.38.0402 22614995

[28] Gascoyne RD Aoun P Wu D Chhanabhai M Skinnider BF Greiner TC. Prognostic significance of anaplastic lymphoma kinase (Alk) protein expression in adults with anaplastic Large cell lymphoma. Blood J Of Am Soc Of Hematol (1999) 93(11):3913–21. 10339500

[29] Maurer MJ Ellin F Srour L Jerkeman M Bennani NN Connors JM. International assessment of event-free survival At 24 months and subsequent survival in peripheral T-cell lymphoma. J Of Clin Oncol Off J Of Am Soc Of Clin Oncol (2017) 35(36):4019–26. doi: 10.1200/JCO.2017.73.8195 PMC5736237 29072976

[30] Savage KJ Harris NL Vose JM Ullrich F Jaffe ES Connors JM. Alk– anaplastic Large-cell lymphoma is clinically and immunophenotypically different from both alk alcl and peripheral T-cell lymphoma, not otherwise specified: Report from the international peripheral T-cell lymphoma project. Blood J Of Am Soc Of Hematol (2008) 111(12):5496–504. doi: 10.1182/blood-2008-01-134270 18385450

[31] Werner MT Zhao C Zhang Q Wasik MA. Nucleophosmin-anaplastic lymphoma kinase: The ultimate oncogene and therapeutic target. Blood J Of Am Soc Of Hematol (2017) 129(7):823–31. doi: 10.1182/blood-2016-05-717793 27879258

[32] Morris SW Kirstein MN Valentine MB Dittmer KG Shapiro DN Saltman DL. Fusion of a kinase gene, alk, to a nucleolar protein gene, npm, in non-hodgkin's lymphoma. Sci (New York N.Y.) (1994) 263(5151):1281–4. doi: 10.1126/science.8122112 8122112

[33] Lobello C Tichy B Bystry V Radova L Filip D Mraz M. Stat3 and Tp53 mutations associate with poor prognosis in anaplastic Large cell lymphoma. Leukemia (2021) 35(5):1500–5. doi: 10.1038/s41375-020-01093-1 PMC8102183 33247178

[34] Larose H Prokoph N Matthews JD Schlederer M Hogler S Alsulami AF. Whole exome sequencing reveals Notch1 mutations in anaplastic Large cell lymphoma and points to notch both as a key pathway and a potential therapeutic target. Haematologica (2021) 106(6):1693–704. doi: 10.3324/haematol.2019.238766 PMC8168516 32327503

[35] Parrilla Castellar ER Jaffe ES Said JW Swerdlow SH Ketterling RP Knudson RA. Alk-negative anaplastic Large cell lymphoma is a genetically heterogeneous disease with widely disparate clinical outcomes. Blood J Of Am Soc Of Hematol (2014) 124(9):1473–80. doi: 10.1182/blood-2014-04-571091 PMC4148769 24894770

[36] Luchtel RA Dasari S Oishi N Pedersen MB Hu G Rech KL. Molecular profiling reveals immunogenic cues in anaplastic Large cell lymphomas with Dusp22 rearrangements. Blood J Of Am Soc Of Hematol (2018) 132(13):1386–98. doi: 10.1182/blood-2018-03-838524 PMC6161771 30093402

[37] King RL Dao LN Mcphail ED Jaffe ES Said J Swerdlow SH. Morphologic features of alk-negative anaplastic Large cell lymphomas with Dusp22 rearrangements. Am J Of Surg Pathol (2016) 40(1):36–43. doi: 10.1097/PAS.0000000000000500 26379151 PMC4834837

[38] Hapgood G Ben-Neriah S Mottok A Lee DG Robert K Villa D. Identification of high-risk Dusp22-rearranged alk-negative anaplastic Large cell lymphoma. Br J Of Haematology (2019) 186(3):E28–31. doi: 10.1111/bjh.15860 PMC7679007 30873584

[39] Fitzpatrick MJ Massoth LR Marcus C Vergilio J Severson E Duncan D. Jak2 rearrangements are a recurrent alteration in Cd30 systemic T-cell lymphomas with anaplastic morphology. Am J Of Surg Pathol (2021) 45(7):895–904. doi: 10.1097/PAS.0000000000001708 34105517

[40] Miranda RN Aladily TN Prince HM Kanagal-Shamanna R De Jong D Fayad LE. Breast implant-associated anaplastic Large-cell lymphoma: Long-term follow-up of 60 patients. J Of Clin Oncol Off J Of Am Soc Of Clin Oncol (2014) 32(2):114–20. doi: 10.1200/JCO.2013.52.7911 PMC4062709 24323027

[41] Oishi N Brody GS Ketterling RP Viswanatha DS He R Dasari S. Genetic subtyping of breast implant–associated anaplastic Large cell lymphoma. Blood J Of Am Soc Of Hematol (2018) 132(5):544–7. doi: 10.1182/blood-2017-12-821868 PMC6073323 29921615

[42] Hu H Johani K Almatroudi A Vickery K Van Natta B Kadin ME. Bacterial biofilm infection detected in breast implant–associated anaplastic Large-cell lymphoma. Plast And Reconstructive Surg (2016) 137(6):1659–69. doi: 10.1097/PRS.0000000000002010 26890506

[43] Di Napoli A Jain P Duranti E Margolskee E Arancio W Facchetti F. Targeted next generation sequencing of breast implant-associated anaplastic Large cell lymphoma reveals mutations in Jak/Stat signalling pathway genes, Tp53 and Dnmt3a. Br J Of Haematology (2018) 180(5):741–4. doi: 10.1111/bjh.14431 27859003

[44] Jung KS Cho S Kim SJ Ko YH Kim WS. Clinical features and treatment outcome of Epstein–Barr virus-positive nodal T-cell lymphoma. Int J Of Hematol (2016) 104(5):591–5. doi: 10.1007/s12185-016-2068-1 27456462

[45] Wai CMM Chen S Fan S Leong SM Zheng W Low LCY. Immune pathway upregulation and lower genomic instability distinguish ebv-positive nodal T/Nk-cell lymphoma from enktl and ptcl-nos. Haematologica (2022) 107(8):1864–79. doi: 10.3324/haematol.2021.280003 PMC9335103 35021606

[46] Epstein-Peterson ZD Horwitz SM. Molecularly targeted therapies for relapsed and refractory peripheral T-cell lymphomas. In: Seminars in hematology. Elsevier (2021). p. 78–84. 10.1053/j.seminhematol.2021.02.004 PMC8496899 33906725

[47] Couronné L Bastard C Bernard OA. Tet2 and Dnmt3a mutations in human T-cell lymphoma. New Engl J Of Med (2012) 366(1):95–6. doi: 10.1056/NEJMc1111708 22216861

[48] Dhiran K Libien J Brunson C Jain P Gupta R. Ezh2 expression is increased in neoplastic T cells as compared with reactive lymphocytes and differs in different subtypes of T-cell lymphoma. Am J Of Clin Pathol (2012) 138(Suppl_1):A161. doi: 10.1093/ajcp/138.suppl1.147

[49] Ishitsuka K Izutsu K Maruyama D Makita S Jacobsen E Horwitz S. First-In-Human study of the Ezh1 and Ezh2 dual inhibitor valemetostat tosylate (Ds-3201b) in patients with relapsed or refractory non-Hodgkin lymphomas. Hematological Oncol (2021) 39. doi: 10.1002/hon.14_2879

[50] Foss FM Porcu P Horwitz SM Izutsu K Ishitsuka K Kato K. A global phase 2 study of valemetostat tosylate (Valemetostat) in patients with relapsed or refractory (R/R) peripheral T-cell lymphoma (Ptcl), including R/R adult T-cell Leukemia/Lymphoma (Atl)-Valentine-Ptcl01. Blood (2021) 138:2533. doi: 10.1182/blood-2021-144676

[51] Park J Jong H Kim SG Jung Y Lee K Lee J. Inhibitors of histone deacetylases induce tumor-selective cytotoxicity through modulating aurora-a kinase. J Of Mol Med (2008) 86(1):117–28. doi: 10.1007/s00109-007-0260-8 17851643

[52] Coiffier B Pro B Prince HM Foss F Sokol L Greenwood M. Results from a pivotal, open-label, phase ii study of romidepsin in relapsed or refractory peripheral T-cell lymphoma after prior systemic therapy. J Of Clin Oncol (2012) 30(6):631–6. doi: 10.1200/JCO.2011.37.4223 22271479

[53] O'connor OA Horwitz S Masszi T Van Hoof A Brown P Doorduijn J. Belinostat in patients with relapsed or refractory peripheral T-cell lymphoma: Results of the pivotal phase ii belief (Cln-19) study. J Of Clin Oncol Off J Of Am Soc Of Clin Oncol (2015) 33(23):2492–9. doi: 10.1200/JCO.2014.59.2782 PMC5087312 26101246

[54] Delarue R Dupuis J Sujobert P Barbieux S Marçais A Tournilhac O. Treatment with hypomethylating agent 5-azacytidine induces sustained response in angioimmunoblastic T cell lymphomas. Blood (2016) 128(22):4164. doi: 10.1182/blood.V128.22.4164.4164

[55] Yoon SE Cho J Kim YJ Kim SJ Kim WS. Real-world efficacy of 5-azacytidine as salvage chemotherapy for angioimmunoblastic T-cell lymphoma. Clin Lymphoma Myeloma And Leukemia (2022) 22(11):E972–80. Invalid Date. doi: 10.1016/j.clml.2022.07.009 35995702

[56] Horwitz SM Moskowitz AJ Jacobsen ED Mehta-Shah N Khodadoust MS Fisher DC. The combination of duvelisib, a Pi3k-Δ, Γ inhibitor, and romidepsin is highly active in Relapsed/Refractory peripheral T-cell lymphoma with low rates of transaminitis: Results of parallel multicenter, phase 1 combination studies with expansion cohorts. Blood (2018) 132:683. doi: 10.1182/blood-2018-99-115241

[57] Kalac M Jain S Tam CS Goldfinger M Xiao Z Montanari F. A real world experience of combined treatment with romidepsin and azacitidine in patients with peripheral T-cell lymphoma-a bridge to transplant and an effective salvage for the unfit. Blood (2022) 140(Supplement 1):9422–4. doi: 10.1182/blood-2022-165165

[58] Ruan J Moskowitz AJ Mehta-Shah N Sokol L Chen Z Rahim R. Multi-center phase ii study of oral azacitidine (Cc-486) plus chop as initial treatment for peripheral T-cell lymphoma (Ptcl). Blood (2020) 136:33–4. doi: 10.1182/blood-2020-136023

[59] Yamagishi M Hori M Fujikawa D Ohsugi T Honma D Adachi N. Targeting excessive Ezh1 and Ezh2 activities for abnormal histone methylation and transcription network in malignant lymphomas. Cell Rep (2019) 29(8):2321–37,e7. doi: 10.1016/j.celrep.2019.10.083 31747604

[60] Hu L Zhang X Li H Lin S Zang S. Targeting Tet2 as a therapeutic approach for angioimmunoblastic T cell lymphoma. Cancers (2022) 14(22):5699. doi: 10.3390/cancers14225699 36428791 PMC9688210

[61] Guo J Zhang R Yang Z Duan Z Yin D Zhou Y. Biological roles and therapeutic applications of Idh2 mutations in human cancer. Front In Oncol (2021) 11:644857. doi: 10.3389/fonc.2021.644857 PMC8107474 33981605

[62] Sakata-Yanagimoto M. Multistep tumorigenesis in peripheral T cell lymphoma. Int J Of Hematol (2015) 102(5):523–7. doi: 10.1007/s12185-015-1738-8 25628103

[63] Fujisawa M Sakata-Yanagimoto M Nishizawa S Komori D Gershon P Kiryu M. Activation of rhoa–Vav1 signaling in angioimmunoblastic T-cell lymphoma. Leukemia (2018) 32(3):694–702. doi: 10.1038/leu.2017.273 28832024 PMC5843900

[64] Huang D Song TL Nairismägi M Laurensia Y Pang W Zhe DCM. Evaluation of the Pik3 pathway in peripheral T-cell lymphoma and Nk/T-cell lymphoma. Br J Of Haematology (2020) 189(4):731–44. doi: 10.1111/bjh.16435 PMC7322801 32004387

[65] Horwitz SM Koch R Porcu P Oki Y Moskowitz A Perez M. Activity of the Pi3k-Δ, Γ inhibitor duvelisib in a phase 1 trial and preclinical models of T-cell lymphoma. Blood J Of Am Soc Of Hematol (2018) 131(8):888–98. 10.1182/blood-2017-08-802470 PMC5824337 29233821

[66] Pro B Brammer JE Casulo C Jacobsen E Mead M Mehta-Shah N. Duvelisib in patients with Relapsed/Refractory peripheral T-cell lymphoma from the phase 2 primo trial: Dose optimization efficacy update and expansion phase initial results. Blood (2020) 136:38–9. doi: 10.1182/blood-2020-140412

[67] Jacobsen ED Zinzani PL Zain J Mead M Casulo C Gritti G. Duvelisib in patients with Relapsed/Refractory peripheral T-cell lymphoma from the phase 2 primo trial expansion phase: Impact of prior treatment and expanded safety analysis. Blood 140(Supplement 1) (2022), 9387–9. doi: 10.1182/blood-2022-169432

[68] Huen A Haverkos BM Zain J Radhakrishnan R Lechowicz MJ Devata S. Phase I/Ib study of tenalisib (Rp6530), a dual Pi3k Δ/Γ inhibitor in patients with Relapsed/Refractory T-cell lymphoma. Cancers (2020) 12(8):2293. doi: 10.3390/cancers12082293 32824175 PMC7463651

[69] Horwitz S Moskowitz A Mehta-Shah N Jacobsen E Khodadoust M Ganesan N. The combination of duvelisib and romidepsin (Dr) is highly active against Relapsed/Refractory peripheral T-cell lymphoma with low rates of transaminitis: Final results. Hematological Oncol (2021) 39. doi: 10.1002/hon.56_2879

[70] Moskowitz AJ Jacobsen E Ruan J Schatz JH Obadi O Motylinski K. Durable responses observed with jak inhibition in T-cell lymphomas. Blood (2018) 132:2922. doi: 10.1182/blood-2018-99-112123

[71] Leveille E Chan LN Mirza A Kume K Müschen M. Syk and Zap70 kinases in autoimmunity and lymphoid malignancies. Cell Signalling (2022), 110331. doi: 10.1016/j.cellsig.2022.110331 35398488

[72] Horwitz SM Feldman TA Hess BT Khodadoust MS Kim YH Munoz J. The novel Syk/Jak inhibitor cerdulatinib demonstrates good tolerability and clinical response in a phase 2a study in Relapsed/Refractory peripheral T-cell lymphoma and cutaneous T-cell lymphoma. Blood (2018) 132:1001. doi: 10.1182/blood-2018-99-119944

[73] Qi W Spier C Liu X Agarwal A Cooke LS Persky DO. Alisertib (Mln8237) an investigational agent suppresses aurora a and b activity, inhibits proliferation, promotes endo-reduplication and induces apoptosis in T-nhl cell lines supporting its importance in ptcl treatment. Leukemia Res (2013) 37(4):434–9. doi: 10.1016/j.leukres.2012.10.017 PMC3816641 23153524

[74] O'connor OA Ozcan M Jacobsen ED Roncero JM Trotman J Demeter J. Randomized phase iii study of alisertib or investigator's choice (Selected single agent) in patients with relapsed or refractory peripheral T-cell lymphoma. J Of Clin Oncol (2019). doi: 10.1200/JCO.18.00899 PMC6494247 30707661

[75] Merino M Kasamon Y Li H Ma L Leong R Zhou J. Fda approval summary: Crizotinib for pediatric and young adult patients with relapsed or refractory systemic anaplastic Large cell lymphoma. Pediatr Blood Cancer (2022):E29602. doi: 10.1002/pbc.29602 35561013

[76] Gambacorti Passerini C Farina F Stasia A Redaelli S Ceccon M Mologni L. Crizotinib in advanced, chemoresistant anaplastic lymphoma kinase–positive lymphoma patients. J Of Natl Cancer Institute (2014) 106(2):Djt378. doi: 10.1093/jnci/djt378 24491302

[77] Richly H Kim TM Schuler M Kim D Harrison SJ Shaw AT. Ceritinib in patients with advanced anaplastic lymphoma kinase–rearranged anaplastic Large-cell lymphoma. Blood J Of Am Soc Of Hematol (2015) 126(10):1257–8. doi: 10.1182/blood-2014-12-617779 PMC4559938 26337354

[78] Hudson S Wang D Middleton F Nevaldine BH Naous R Hutchison RE. Crizotinib induces apoptosis and gene expression changes in alk anaplastic Large cell lymphoma cell lines; brentuximab synergizes and doxorubicin antagonizes. Pediatr Blood Cancer (2018) 65(8):E27094. doi: 10.1002/pbc.27094 29697184

[79] Lunning MA Horwitz S. Treatment of peripheral T-cell lymphoma: Are we data driven or driving the data? Curr Treat Options In Oncol (2013) 14(2):212–23. doi: 10.1007/s11864-013-0232-x PMC3938283 23568456

[80] Fanale MA Horwitz SM Forero-Torres A Bartlett NL Advani RH Pro B. Brentuximab vedotin in the front-line treatment of patients with Cd30+ peripheral T-cell lymphomas: Results of a phase I study. J Of Clin Oncol Off J Of Am Soc Of Clin Oncol (2014) 32(28):3137–43. doi: 10.1200/JCO.2013.54.2456 PMC4171358 25135998

[81] Horwitz S O'connor O Pro B Trümper L Iyer S Advani R. The echelon-2 trial: 5-year results of a randomized, phase iii study of brentuximab vedotin with chemotherapy for Cd30-positive peripheral T-cell lymphoma. Ann Of Oncol (2022) 33(3):288–98. doi: 10.1016/j.annonc.2021.12.002 PMC9447792 34921960

[82] Murai K Hiroyuki H Sato A Yasuro M Mori Y Sakuma T. Mogamulizumab monotherapy in the treatment of Relapsed/Refractory peripheral T-cell lymphoma or cutaneous T-cell lymphoma patients in single-institution experience. Blood (2017) 130:5179.

[83] Ishida T Jo T Takemoto S Suzushima H Uozumi K Yamamoto K. Dose-intensified chemotherapy alone or in combination with mogamulizumab in newly diagnosed aggressive adult T-cell leukaemia-lymphoma: A randomized phase ii study. Br J Of Haematology (2015) 169(5):672–82. doi: 10.1111/bjh.13338 PMC5024033 25733162

[84] Karube K Aoki R Nomura Y Yamamoto K Shimizu K Yoshida S. Usefulness of flow cytometry for differential diagnosis of precursor and peripheral T-cell and nk-cell lymphomas: Analysis of 490 cases. Pathol Int (2008) 58(2):89–97. doi: 10.1111/j.1440-1827.2007.02195.x 18199158

[85] Hamadani M Collins GP Caimi PF Samaniego F Spira A Davies A. Camidanlumab tesirine in patients with relapsed or refractory lymphoma: A phase 1, open-label, multicentre, dose-escalation, dose-expansion study. Lancet Haematology (2021) 8(6):E433–45. doi: 10.1016/S2352-3026(21)00103-4 PMC9241579 34048682

[86] Zain J Simpson J Palmer J Wong J Dandapani S Colcher D. Phase I study of yttrium-90 labeled anti-Cd25 (Atac) monoclonal antibody plus beam for autologous hematopoietic cell transplantation (Ahct) in patients with mature T-cell non-Hodgkin lymphoma, the “A-Tac-Beam regimen”. Blood (2018) 132:611. doi: 10.1182/blood-2018-99-116907

[87] Cai Q Huang H Bai B Yan G Li J Lin S. The serum spectrum of cytokines in patients with Nk/T-cell lymphoma and its cilincial significance in survival. Blood (2013) 122(21):1759. doi: 10.1182/blood.V122.21.1759.1759

[88] Fink EC Ebert BL. The novel mechanism of lenalidomide activity. Blood J Of Am Soc Of Hematol (2015) 126(21):2366–9. doi: 10.1182/blood-2015-07-567958 PMC4653765 26438514

[89] Toumishey E Prasad A Dueck G Chua N Finch D Johnston J. Final report of a phase 2 clinical trial of lenalidomide monotherapy for patients with T-cell lymphoma. Cancer (2015) 121(5):716–23. doi: 10.1002/cncr.29103 25355245

[90] Morschhauser F Fitoussi O Haioun C Thieblemont C Quach H Delarue R. A phase 2, multicentre, single-arm, open-label study to evaluate the safety and efficacy of single-agent lenalidomide (Revlimid®) in subjects with relapsed or refractory peripheral T-cell non-Hodgkin lymphoma: The expect trial. Eur J Of Cancer (2013) 49(13):2869–76. doi: 10.1016/j.ejca.2013.04.029 23731832

[91] Lunning MA Horwitz SM Advani R Vose JM Lee HJ Mehta-Shah N. Phase I/Ii study of choep plus lenalidomide as initial therapy for patients with stage ii-iv peripheral T-cell lymphoma: Phase ii results. Blood (2018) 132:2899. doi: 10.1182/blood-2018-99-114110 PMC11220724 37217196

[92] Mehta-Shah N Lunning MA Boruchov AM Ruan J Nair S Lynch P. No title. a phase I/Ii trial of the combination of romidepsin and lenalidomide in patients with Relapsed/Refractory lymphoma and myeloma: Activity in T-cell lymphoma. Am J Hematol (2015) 96(10):1211–22. doi: 10.1200/jco.2015.33.15_suppl.8521

[93] Mehta-Shah N Moskowitz AJ Lunning MA Lynch P Scheuerman M Minnal V. A phase Ib/Iia trial of the combination of romidepsin, lenalidomide and carfilzomib in patients with Relapsed/Refractory lymphoma shows complete responses in relapsed and refractory b-and T-cell lymphomas. Blood (2017) 130:821. doi: 10.1182/blood.V130.Suppl_1.821.821

[94] Ratner L Waldmann TA Janakiram M Brammer JE. Rapid progression of adult T-cell leukemia–lymphoma after pd-1 inhibitor therapy. New Engl J Of Med (2018) 378(20):1947–8. doi: 10.1056/NEJMc1803181 29768155

[95] Khodadoust M Rook AH Porcu P Foss FM Moskowitz AJ Shustov AR. Pembrolizumab for treatment of Relapsed/Refractory mycosis fungoides and sezary syndrome: Clinical efficacy in a citn multicenter phase 2 study. Blood (2016) 128(22):181. doi: 10.1182/blood.V128.22.181.181

[96] Jain S Van Scoyk A Morgan EA Matthews A Stevenson K Newton G. Targeted inhibition of Cd47-sirpα requires fc-fcγr interactions to maximize activity in T-cell lymphomas. Blood J Of Am Soc Of Hematol (2019) 134(17):1430–40. 10.1182/blood.2019001744 PMC6839960 31383641

[97] Safarzadeh Kozani P Safarzadeh Kozani P Rahbarizadeh F. Car-T cell therapy in T-cell malignancies: Is success a low-hanging fruit? Stem Cell Res Ther (2021) 12(1):1–17. doi: 10.1186/s13287-021-02595-0 34620233 PMC8499460

[98] Wu Y Chen D Lu Y Dong S Ma R Tang W. A new immunotherapy strategy targeted Cd30 in peripheral T-cell lymphomas: Car-modified T-cell therapy based on Cd30 mab. Cancer Gene Ther (2022) 29(2):167–77. doi: 10.1038/s41417-021-00295-8 PMC8850188 33514882

[99] Spinner S Crispatzu G Yi J Munkhbaatar E Mayer P Höckendorf U. Re-activation of mitochondrial apoptosis inhibits T-cell lymphoma survival and treatment resistance. Leukemia (2016) 30(7):1520–30. doi: 10.1038/leu.2016.49 27055871

[100] Koch R Christie AL Crombie JL Palmer AC Plana D Shigemori K. Biomarker-driven strategy for Mcl1 inhibition in T-cell lymphomas. Blood J Of Am Soc Of Hematol (2019) 133(6):566–75. doi: 10.1182/blood-2018-07-865527 PMC6367646 30498064

[101] Gualberto A Scholz C Mishra V Janes MR Kessler L. Ps1002 rhoe, Cxcl12 and Cxcr3 may identify complete responses in acute myeloid leukemia patients treated with tipifarnib. Hemasphere (2019) 3(S1):450–1. doi: 10.1097/01.HS9.0000562304.50732.0a

[102] Witzig T Sokol L Kim W Foss F Jacobsen E De La Cruz Vincente F. Tipifarnib in relapsed or refractory angioimmunoblastic T-cell lymphoma (Aitl) and Cxcl12 peripheral T-cell lymphoma (Ptcl): Preliminary results from a phase 2 study. Hematological Oncol (2019) 37:64–5. doi: 10.1002/hon.32_2629

